# The variances in cytokine production profiles from non- or activated THP-1, Kupffer cell and human blood derived primary macrophages following exposure to either alcohol or a panel of engineered nanomaterials

**DOI:** 10.1371/journal.pone.0220974

**Published:** 2019-08-08

**Authors:** Ali Kermanizadeh, David M. Brown, Vicki Stone

**Affiliations:** Heriot Watt University, School of Engineering and Physical Sciences, Nano Safety Research Group, Edinburgh, United Kingdom; Dasman Diabetes Institute, KUWAIT

## Abstract

The portfolio of cytokines is key to the function of macrophages as sentries of the innate immune system as well as being critical for the transition from innate to adaptive immunity. Cytokine bias is critical in the fate of macrophages into a continuum of inflammatory to anti-inflammatory macrophages. Due to advances in the field of toxicology, increasingly advanced multi-cellular *in vitro* safety assessment models are being developed in order to allow for a better predication of potential adverse effects in humans with many of these models include a macrophage population. The selection of the correct macrophage cells in these advanced *in vitro* models is critical for a physiologically relevant and realistic immune response. In this study we investigated cytokine response profile (IL1-β, IL6, IL10 and TNF-α) of activated and non-activated THP-1 (immortalized monocyte-like cell line), primary human Kupffer cells (liver resident macrophages) and human primary peripheral blood mononuclear cells following exposure of a panel of nanomaterials or ethanol. The data demonstrated that the THP-1 cell line are not great cytokine producers. The PBMC appear to be a good *in vitro* surrogate for circulating/pro-inflammatory macrophages but are not a suitable replacement for Kupffer cells. The findings from this study highlight the necessity for the selection of appropriate macrophages populations to meet the specific physiological requirements of *in vitro* experiment.

## Introduction

Macrophages are the sentinels of the immune system and in concert with neutrophils, serve as the first line of defence against viral, bacterial and parasitic infections [1,2]. Additionally, they might be important in clearance of non-protein based particulate matter (including nanomaterials (NMs)). Macrophages can act not only as professional antigen presenting cells (APC), but also have crucial pro- or anti-inflammatory roles, dependent on how they are activated [3,4]. Macrophages are normally in a non-activated state, but can be activated by an assortment of stimuli during the immune response [5]. Generally speaking, phagocytosis provides the initial antigen stimulus for activation of macrophages, but other factors such as cytokines secreted by helper T lymphocytes, including interferon gamma (IFN-ϒ) also heavily contribute to the process. In fact, IFN-ϒ is one of the most effective macrophage activators—(“classically" activated macrophages).

A crucial characteristic of macrophages is their functional plasticity, predominantly with regards to their response based on the inflammatory microenvironment. This response can be generally categorised into “M1”—pro-inflammatory or “M2”—immunoregulatory. The role of macrophage polarization in the governance of a NM-induced immune response is not yet fully understood. As a further complication, macrophages can adapt to their tissue location, and their biological function can be heavily governed by their location within the specific tissue and in all reality the interactions of these cells with NMs are both tissue- and cell-state-specific [6].

Macrophages stand poised to rapidly produce large amounts of inflammatory cytokines in response to danger signals. The production of these cytokines can initiate a cascade of inflammatory mediator release that can lead to wholesale tissue destruction. Hence macrophages are implicated in a wide range of pathological conditions including but not limited to metabolic inflammation [7–9], liver hepatitis, fibrosis and cancer [10], chronic obstructive pulmonary disease [11] and cardiovascular disease [12].

Interleukin (IL)6 is an important mediator of innate immunity. The protein is secreted by macrophages; and instigates various innate immune signalling cascades resulting in an augmentation of inflammatory responses and cell recruitment [13]. Furthermore, the cytokine is responsible for stimulating acute phase protein response from the liver as well as the production of neutrophils [14,15].

Tumour necrosis factor-α (TNF-α) is one of the main contributors to septic shock. They stimulate the release of corticotropic releasing hormone, which induces fever, and suppress appetite. Furthermore, it also stimulates the acute inflammatory response by promoting the synthesis of C-reactive protein and other mediators in the liver. TNF-α also induces vasodilation and loss of vascular permeability both critical for leukocyte infiltration. The cytokine is also important in the recruitment of inflammatory cells to the inflammation site [16].

IL1β is an extremely strong pro-inflammatory cytokine. Similarly to TNF-α, IL1β is an endogenous pyrogen that is produced at the early stages of the immune response to infections and physical stress. During an inflammatory response, IL1β stimulates the production of acute phase proteins from the liver and acts to induce fever and prostaglandin secretion [17].

IL10 is a multi-functional cytokine with diverse effects on a wide range of cells. The cytokine’s principle function is to terminate inflammatory responses [18,19]. The cytokine also plays a crucial role in the differentiation of regulatory T cells, which are involved in the control of inflammatory responses and development of immune tolerance [19].

Nanotechnology promises significant economic and societal benefits, but the commercialization of this technology is partially hindered by safety concerns. Due to the limitations of traditional mono-culture cellular models and the advances in the field of toxicology, increasingly advanced multi-cellular *in vitro* safety assessment models are being developed in order to allow for a better predication of potential adverse effects in humans. Many of these models include a macrophage population (as is the case in European Commission funded project PATROLS project) [20], where multi-cellular *in vitro* 3D models of lung, gastrointestinal tract and liver all incorporate a macrophage population. As suggested above, the selection of the correct macrophage cells is critical for a physiologically relevant and realistic immune response, which would allow for the *in vitro* system to be considered as a better surrogate to animal models. This study was designed to scrutinize the profile of cytokine responses by activated and non-activated THP-1 (immortalized monocyte-like cell line), primary human Kupffer cells (KCs) (liver resident macrophages) and human primary peripheral blood mononuclear cells (PBMC).

In these experiments, the macrophage models were all exposed to a panel of NMs—namely—zinc oxide (ZnO), silver (Ag), titanium dioxide (TiO_2_) and quartz containing ~ 87% crystalline silica (DQ12). These NMs have been extensively studied previously both in *vitro* and *in vivo* [21–26], allowing this study to benchmark the macrophage model responses to existing data. Additionally, ethanol was included as a non-particulate chemical control.

## Materials and methods

### Nanomaterials

The NMs were sourced as follows: TiO_2_ (JRC Nanomaterials Repository—Italy, JRCNM01005a), ZnO (JRC Nanomaterials Repository—Italy, JRCNM01101a), Ag (Fraunhofer IME—Germany, NM300-K) and crystalline silica (Institute of Occupational Medicine—UK, DQ12). The NMs were sub-sampled under Good Laboratory Practice conditions and preserved under argon in the dark until use (with the exception of the DQ12).

### Characterisation of the materials

The investigated particles were characterised using a combination of analytical techniques in order to infer primary physical and chemical properties important for understanding their toxicological behaviour. These measured physical and chemical properties have been described in detail previously [27,28]. Furthermore, the hydrodynamic size distributions of the particles in the different biological media were determined at a concentration of 10 μg/ml at 24 hr by Dynamic Light Scattering (DLS) using a Malvern Metasizer nano series—Nano ZS (USA) (Table 1).

**Table 1:** Main physical and chemical properties of investigated NMs.

NM code	NM type	Phase	Primary size (nm)	Surface area [m^2^/g] (BET)	Known coating	Size in RPMI (nm)^Ψ^	Size in KC medium (nm)^Ψ^
JRCNM01005a	TiO_2_	Rutile-anatase	15–24	46	None	337.47±8.27	427.5±39.2
JRCNM01101a	ZnO	Coated	152	15	Triethoxycaprl-silane	261.1±6.45	287.4±8.72
NM300-K	Ag	-	15	-	4% each of polyoxyethylene glycerol trioleate and Tween 20	35.1±1.05	56.57±3.83
DQ12	Silica	-	-	-	None	256.87±3.24	229.2±22.9

^Ψ^ Size in biological media measured within 30 min of sonication.

### Isolation of primary blood macrophages from buffy coats

Buffy coats (fraction of an anticoagulated blood sample that contains most of the white blood cells and platelets following density gradient centrifugation of the blood) were obtained from the Scottish National Blood Transfusion Service. Buffy coat was prepared to a concentration of 1/4 in PBS, before being distributed equally into four 25 ml falcon tubes. Next, 13 ml of Lymphoprep (Stem Cell Technologies, UK) was pipetted underneath the blood/PBS mixture. The tubes were centrifuged at 1000xg for 20 min at 25°C with slow deceleration. The PBMC layer was removed into two Falcon tubes and made up to 50 ml with PBS. The tubes were again centrifuged for 8 min at 500 g, and the supernatant removed. The cells were re-suspended with a small volume of PBS and pooled. The volume was made up to 50 ml using PBS and a cell count performed. Following a further centrifugation step for 8 min at 500 g, the cell pellet was re-suspended in MACS buffer (Miltenyi Biotech Limited, UK) (0.5% BSA, 2 mM EDTA, PBS and adjusted to pH 7.2) (80 μl—10^7^ total cells) and human CD14 microbeads (Miltenyi Biotech Limited, UK) (10 μl per 10^7^ cells) were mixed well and incubated for 15 min in the fridge. The tube was filled to 50 ml with MACS buffer and centrifuged for 8 min at 500 g. The supernatant was removed and the cell pellet was re-suspended in 500 μl of MACS buffer per 10^8^ cells [29].

The cells were separated using a separation column fitted into a magnetic column (midiMACS separation system—Miltenyi Biotech Limited). The column was primed by flushing with 3 x 3 ml volumes of MACS buffer before the cell suspension was added. The column was washed with 3 x 3 ml volumes of MACS buffer and the unlabelled cells collected. The labelled cells (monocytes) were collected by removing the column from the magnet and flushed with 5ml of MACS buffer. The cells were washed as previously described and adjusted to the required number.

### Cell culture and activation

The THP-1 cells were was obtained from ATCC (USA—ATCC TIB-202D). The THP-1 monocytes were differentiated using 10 ng/ml phorbol 12-myristate 13-acetate (PMA) (Sigma, UK) for 24 hr. The cells were maintained in RPMI (Gibco, UK) medium supplemented with 10% Fetal Calf Serum (FCS) (Gibco, UK), 2 mM L-glutamine, 100 U/ml penicillin/streptomycin (Gibco, UK) at 37°C and 5% CO_2_.

The primary human KCs were purchased from Axol Bioscience Limited (UK). The KCs were thawed in phenol red free Dulbecco's Modified Eagle Medium (DMEM) (Gibco, UK) supplemented with 5% FBS, 2 nM glutamax (Gibco, UK), 15 mM HEPES (Sigma, UK) and 100 U/ml penicillin/streptomycin. The KCs were seeded on type I collagen (Thermo Scientific, UK) coated plates. The liver macrophages were maintained in phenol red free DMEM supplemented with 10% FCS, 1% non-essential amino acids (Sigma, UK), 2 nM glutamax, 15 mM HEPES and 100 U/ml penicillin/streptomycin.

After isolation, the primary blood monocytes were plated in 6 well plates at a concentration of 10^6^ cells /ml (3 ml per well) in RPMI medium supplemented with 10% FCS, 2 mM L-glutamine, 100 U/ml penicillin/streptomycin and incubated at 37°C and 5% CO_2_ for 7 days. After this differentiation step, the plates were placed on ice and washed using ice cold PBS. One millilitre of PBS containing 1 mM EDTA was added to each well and incubated on ice for 30 min. The cells were scraped from the wells using a cell scraper, re-suspended in complete RPMI medium and adjusted to the required concentration prior to plating in 96 well plates.

For the cytokine analysis experiments, all macrophages were utilised at 10^4^ cells per well in a 96 well plate format. For subsequent experiments the macrophage models were either used in a non-activated form, or after activation utilising 20 ng/ml of IFN-ϒ plus 1 μg/ml lipopolysaccharide (LPS) (both Sigma, UK) for 12 hr [30].

### NM dispersion and xenobiotic treatment

NMs were prepared following the NANOGENOTOX dispersion protocol [31]. Following the sonication step, the stock solution of NMs (1 mg/ml) were immediately transferred to ice before being diluted in appropriate complete medium just prior to the experiments. In this experiment, two NM concentrations were used: 5 (Low) and 10 (High) μg/ml (total volume of 100 μl added to each well) as well as negative control (cell culture medium). The two selected NM doses are “relatively low” and were selected based on preliminary data shown to have very little to no cytotoxicity as measured via (WST-1 assay). Additionally, as two exposure groups the macrophages were treated with ethanol at concentrations of 25 (Low) and 50 (High) mM. The ethanol doses were selected as they were shown to have very little toxicity to the macrophages from the preliminary data for this study and the previously published work in hepatocytes [22] yet induced a significant IL8 response from hepatic cell line.

### Cytokine analysis

Following a 24 hr NM and ethanol treatment, the supernatants were collected and stored at -80°C. The levels of human IL1β, IL6, IL10 and TNF-α was determined in the cell supernatant using R&D Systems magnetic Luminex^®^ Performance Assay multiplex kits (bead based immunoassay; Bio-techne, USA) according to the manufacturers instruction. The proteins were detected via a Bio-Rad Bio-Plex MAGPIX multiplex reader. The technology is constructed on the use of analyte-specific antibodies pre-coated onto magnetic microplates embedded with fluorophores at set ratios for each unique microparticle region.

### Statistical analysis

For statistical analysis, the experimental results were compared to their corresponding control values using an ANOVA with Tukey’s multiple comparison. The statistical analysis was carried out utilizing Minitab 18. A p value of <0.05 was considered as significant. All experiments were repeated on three separate occasions.

## Results

### Characteristics of pristine and dispersed NMs

The investigated NMs were extensively characterised by a combination of analytical techniques in order to infer their primary physiochemical properties. A comprehensive description of the measured physical properties for the NMs has been described previously [27,28] and reproduced in Table 1. Furthermore, in order to investigate how the NMs behaved in the different complete cell culture medium, the hydrodynamic size distribution of the materials was investigated (Table 1). Importantly, no endotoxin contamination (≤ 0.25 EU/ml) was detected in any of the NM suspensions [32].

### Cytokine secretion from macrophages following xenobiotic exposure

**Fig 1:**
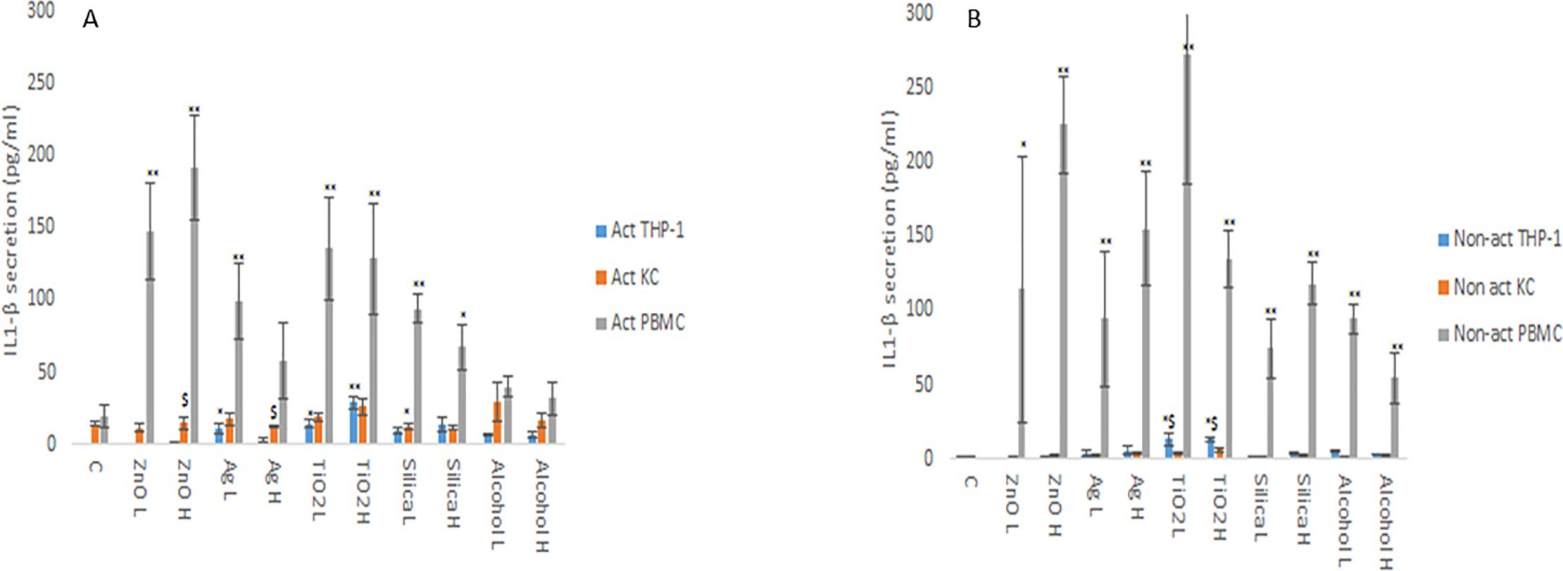
IL1β, secretion from activated and non-activated THP-1, KC and PBMCs exposed to NMs or ethanol. The cells were cultured with cell medium (C) or exposed to the xenobiotic for a period of 24 hr **a)** IL1β - activated macrophages, **b)** IL1β - non-activated macrophages. The values represent mean ± SEM (n = 3) with significance indicated by * p <0.05 and ** p <0.005 of NM-induced effects compared to negative control, $ p <0.05 and $$ p <0.005 signifying statistical differences between the cell types. In order to avoid asymmetry in the statistical distribution of the data the cell population that was clearly different from the others with regards to cytokine secretion levels was excluded in the statistical analysis—i.e. PBMC for IL1-β.

**Fig 2:**
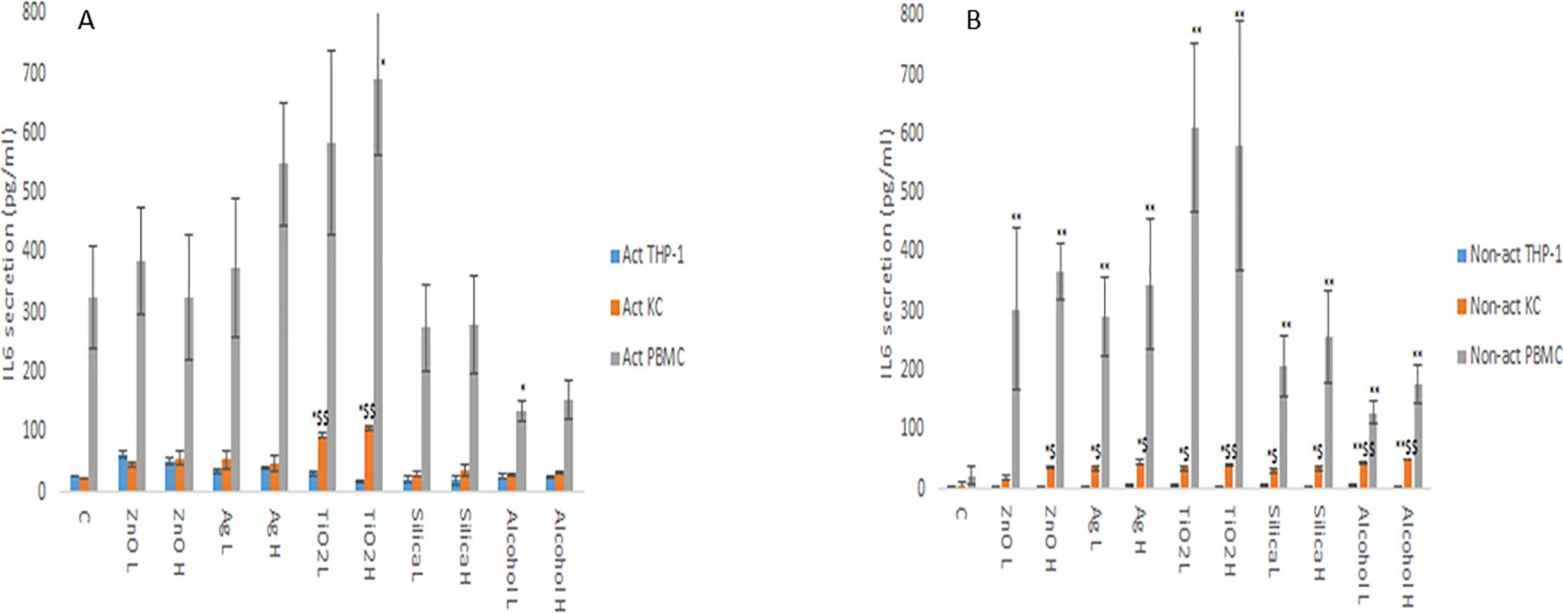
IL6 secretion from activated and non-activated THP-1, KC and PBMCs exposed to NMs or ethanol. The cells were cultured with cell medium (C) or exposed to the xenobiotic for a period of 24 hr **a)** IL6—activated macrophages, **b)** IL6—non-activated macrophages. The values represent mean ± SEM (n = 3) with significance indicated by * p <0.05 and ** p <0.005 of NM-induced effects compared to negative control, $ p <0.05 and $$ p <0.005 signifying statistical differences between the cell types. In order to avoid asymmetry in the statistical distribution of the data the cell population that was clearly different from the others with regards to cytokine secretion levels was excluded in the statistical analysis—i.e. PBMC for IL6.

**Fig 3:**
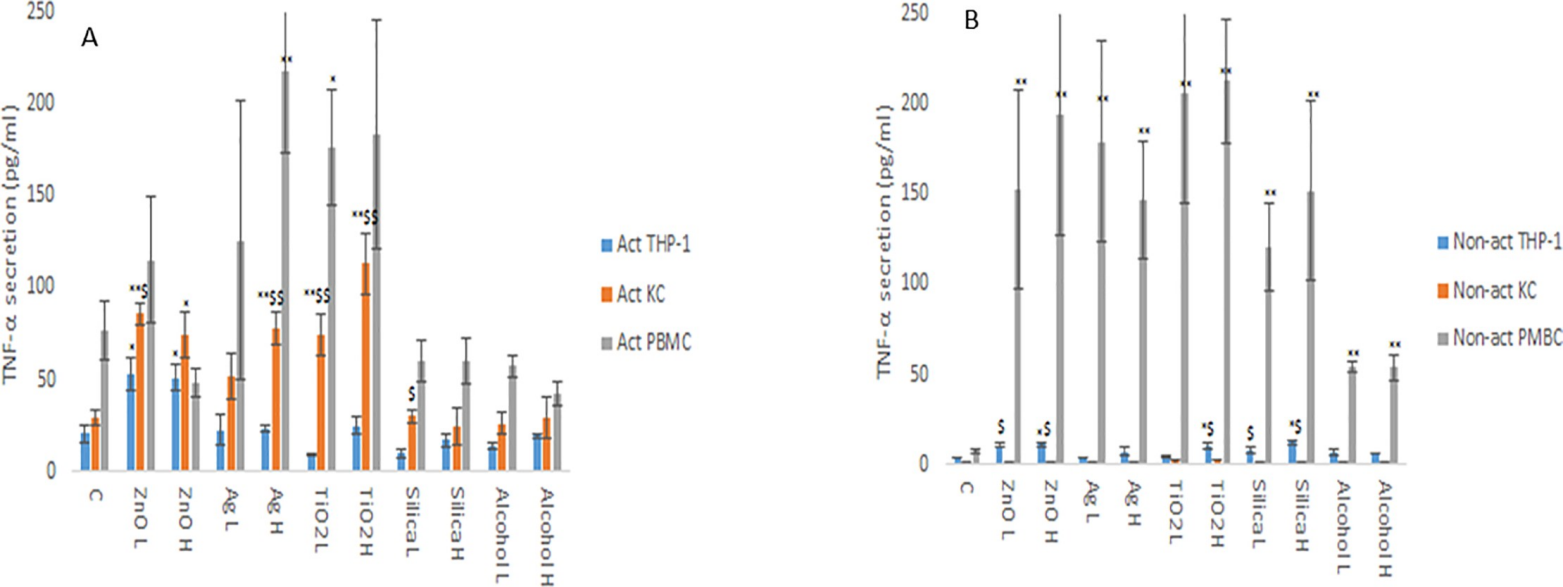
TNF-α secretion from activated and non-activated THP-1, KC and PBMCs exposed to NMs or ethanol. The cells were cultured with cell medium (C) or exposed to the xenobiotic for a period of 24 hr **a)** TNF-α - activated macrophages, **b)** TNF-α - non-activated macrophages. The values represent mean ± SEM (n = 3) with significance indicated by * p <0.05 and ** p <0.005 of NM-induced effects compared to negative control, $ p <0.05 and $$ p <0.005 signifying statistical differences between the cell types. In order to avoid asymmetry in the statistical distribution of the data the cell population that was clearly different from the others with regards to cytokine secretion levels was excluded in the statistical analysis—i.e. PBMC for TNF-α.

**Fig 4:**
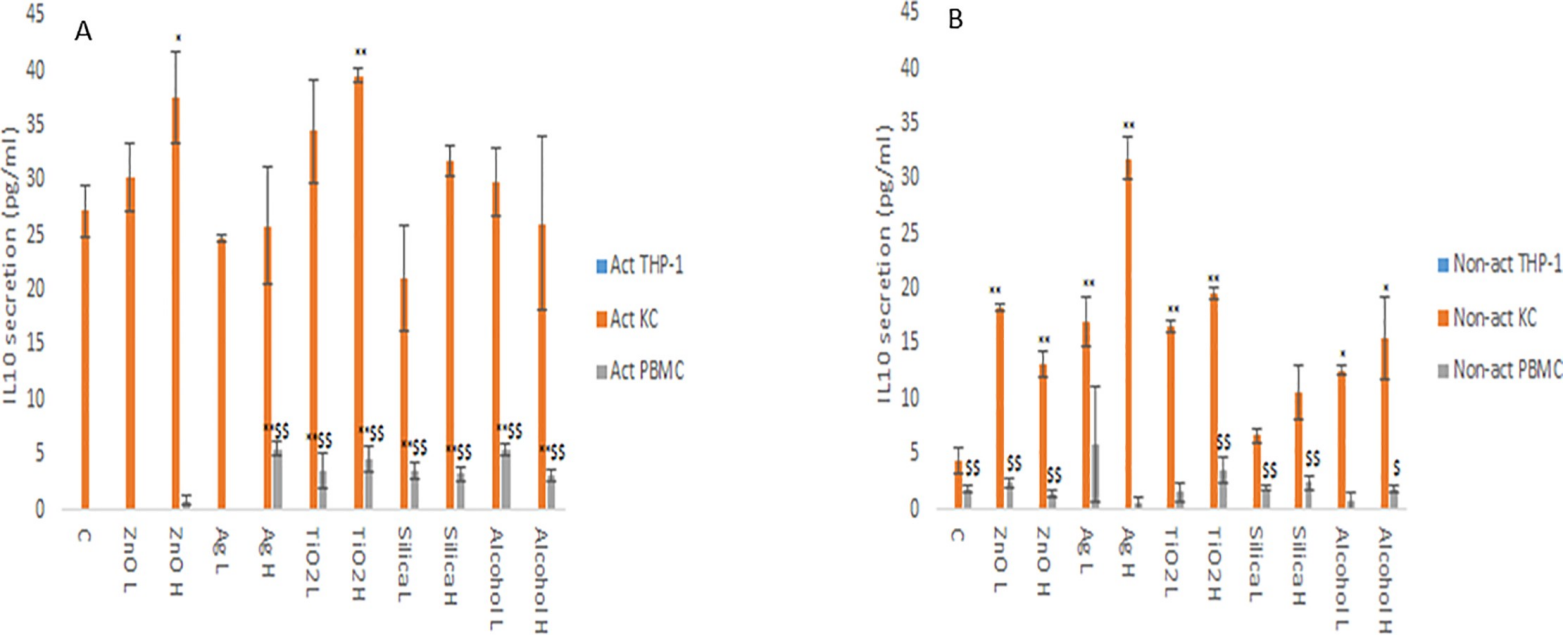
IL10 secretion from activated and non-activated THP-1, KC and PBMCs exposed to NMs or ethanol. The cells were cultured with cell medium (C) or exposed to the xenobiotic for a period of 24 hr **a)** IL10—activated macrophages, **b)** IL10—non-activated macrophages. The values represent mean ± SEM (n = 3) with significance indicated by * p <0.05 and ** p <0.005 of NM-induced effects compared to negative control, $ p <0.05 and $$ p <0.005 signifying statistical differences between the cell types. In order to avoid asymmetry in the statistical distribution of the data the cell population that was clearly different from the others with regards to cytokine secretion levels was excluded in the statistical analysis—i.e. KC for IL10.

The changes in cytokine production levels (IL1-β, IL6, IL10 and TNF-α) as a consequence of NM and ethanol exposure was assessed within the supernatant of non-activated and activated macrophage models. From the data, it is evident that the cytokine profile (both in terms of levels of cytokines produced and the protein itself) of the three macrophage models are markedly and significantly different from each other (Figs 1–4). The data demonstrated that the PBMC secreted the largest quantities of IL1-β, IL6 and TNF-α following NM or ethanol exposure (Figs 1–3). The same pattern was observed for both non-activated and activated macrophages. Overall, the data demonstrated only small variations in the individual cytokine responses between activated and non-activated macrophages exposed to the same xenobiotic. The control levels of cytokine expression in the non-activated models was expectedly lower as compared for the activated counterparts. As suspected, the IL10 secretion levels was highest for the KCs. However, small but significant levels of the anti-inflammatory IL10 cytokine were produced by the PMBC population following NM and ethanol treatment (Fig 4). The IL10 response was entirely absent from THP-1 cells. In fact, generally speaking, the cytokine secretion for the THP-1 was relatively low (although statistical significant was reached for IL1β and TNF-α for the non- or activated THP-1 cells. Interestingly, no distinctly clear differences in the cytokine section patterns between the different NMs was apparent.

## Discussion

Cytokines are powerful signalling molecules that are as crucial to everyday life. They are small proteins that mediate inter/intracellular communication and are produced by numerous cell types but predominately those belonging to the immune system. They coordinate a variety of important processes ranging from the regulation of local and systemic inflammation, chemotaxis, metabolism and tissue repair.

The binding of cytokines to specific cell surface receptors instigate a cell-signalling cascade that orchestrates cell function. This may result in secretion of other cytokines, as well as an increase in the number of surface receptors for other cytokines or in the suppression of the biological response. Cytokines are fundamental to the functions of macrophages, governing the unleashing of an effective immune response and most crucially link an innate and adaptive immune response [33]. Another important feature of cytokine biology is that of functional redundancy: the fact that the different cytokines share similar functions. To this end, it is almost impossible to generalize the effect of a particular cytokine [1]. Furthermore, it is currently extremely difficult to quantify the levels of cytokine required for a biological response *in vivo* as many factors govern the overall efficacy for a given cytokine (i.e. affinity of binding given cytokine for specific receptor, *c*ompetition of ligand-binding, etc. [33–35].

In this study, we compared a range of *in vitro* macrophage models in order to assess their responsiveness to a range of xenobiotics, including NMs and alcohol. The results demonstrate the different macrophage models varied significantly in terms of levels of cytokines produced following a 24 hr xenobiotic exposure. In particular, they differed with regards to the nature of the immune response i.e. whether it was indicative of a pro- or anti-inflammatory response. The data demonstrates that the THP-1 cell line are not great cytokine producers. The PBMC appear to be a great *in vitro* surrogate for circulating/pro-inflammatory macrophages but are not a suitable replacement for hepatic sourced KCs.

It was clear that the PMBCs produced the highest levels of pro-inflammatory cytokines (e.g. IL1β, IL6 and TNF-α), while the KCs secreted sizeable quantities of anti-inflammatory IL10. With respect to the THP-1 cells, these were not effective cytokine producers under the investigated conditions. These findings are similar to a previous examination showing THP-1 being poor cytokine producers as compared to primary macrophages following exposure to carbon nanotubes [36].

It might be important to state that a note of caution is required in the interpretation of findings in this study an increased cytokine secretion by a cell population *in vitro* might not necessarily equate to an inflammatory response *in vivo*. This being said, a number of previous studies have shown good correlation of macrophage cytokine secretion responses *in vitro* and *in vivo* post NM exposure [37–39]. Furthermore, it is almost impossible to ascertain actual cytokine concentrations that would cause a biological response *in vivo*. Moreover, biological responses are rarely limited to a single organ, and cross-talk between different cell types and organs is essential in a biological response to a toxic challenge.

Interestingly, the pre-activation of the macrophages did not greatly influence the inflammatory response for the xenobiotic exposed macrophages. On occasion, the cytokine secretion was higher for the non-activated macrophages exposed to a given xenobiotic as compared to the activated macrophages of the same group. A final noteworthy observation and as touched upon, was the lack of clear patterns in NM or concentration dependant cytokine response under the experimental confounds. This could potentially be explained by the low concentrations of xenobiotic utilised or the specific time-point investigated. Finally, ethanol treatment was not associated with a dose response for any of the cytokines investigated although distinct variances between the different macrophage types was evident which is clearly indicative of the necessity for the appropriate selection of macrophage populations is not a particulate specific issue.

## Conclusion

Overall, the findings from this investigation in addition to the complexities of cytokine biology discussed above, highlight the importance of the selection of appropriate macrophage cell population for a physiologically relevant *in vitro* multi-cellular test systems. As an example, PBMCs are not an appropriate cell type to be used for *in vitro* models of the liver, while the THP-1 cell line is not a good surrogate for primary human PBMCs. The observations from this study are important and highly relevant for the European funded PATROLS project, in which next generation physiologically relevant multi-cellular *in vitro* models of the lung, gastrointestinal tract and liver models are being developed, with the intension to allow improved predictors of *in vivo* toxicological outcomes.

## References

[1] Arango Duque G, Descoteaux A. Macrophage cytokines: involvement in immunity and infectious diseases. Frontiers in Immunology 2014; 7: 491. 10.3389/fimmu.2014.00491 PMC4188125 25339958

[2] Mosser DM, Edwards JP. Exploring the full spectrum of macrophage activation. Nature Reviews Immunology 2008; 8: 958–969. 10.1038/nri2448 19029990 PMC2724991

[3] Benoit M, Desnues B, Mege J. Macrophage polarization in bacterial infections. Journal of Immunology 2008; 181: 3733–3739. 10.4049/jimmunol.181.6.3733 18768823

[4] Franken L, Schiwon M, Kurts C. Macrophages: sentinels and regulators of the immune system. Cellular Microbiology 2016; 18: 475–487. 10.1111/cmi.12580 26880038

[5] Bozza F, Bozza P. Cytokine profiles as markers of disease severity in sepsis: a multiplex analysis. Critical Care 2007; 11: 49. 10.1186/cc5783 PMC2206478 17448250

[6] MacParland SA, Tsoi KM, Ouyang B, Ma XZ, Manuel J, Fawaz A, et al Phenotype determines nanoparticle uptake by human macrophages from liver and blood. ACS Nano 2017; 11: 2428–2443. 10.1021/acsnano.6b06245 28040885

[7] Ahmad R, Al-Roub A, Kouchumon S, Akther N, Thomas R, Kumari M, et al The synergy between palmitate and TNF-α for CCL2 production is dependent on the TRIF/IRF3 pathway: implications for metabolic inflammation. Journal of Immunology 2018; 200: 3599–3611 10.4049/jimmunol.1701552 PMC5937214 29632147

[8] Kouchumon S, Wilson A, Chandy B, Shenouda S, Tuomilehto J, Sindu S, et al Palmitate activates CCL4 expression in human monocytic cells via TLR4/MyD88 dependent activation of NF-κB/MAPK/ PI3K signaling systems. Cellular Physiology and Biochemistry 2018; 46: 953–964. 10.1159/000488824 29669317

[9] Al-Rashed F, Ahmad Z, Iskandar MA, Tuomilehto J, Al-Mulla F, Ahmad R. TNF-α Induces a Pro-Inflammatory Phenotypic Shift in Monocytes through ACSL1: Relevance to Metabolic Inflammation. Cellular Physiology and Biochemistry 2019; 52: 397–407. 10.33594/000000028 30845379

[10] Krenkel O, Tacke F. 2017. Liver macrophages in tissue homeostasis and disease. Nature Reviews Immunology 2017; 306–321. 10.1038/nri.2017.11 28317925

[11] Cruz T, Lopez-Giraldo A, Noell G, Casa-Recasens S, Garcia T, Molins L, et al Multi-level immune response network in mild-moderate Chronic Obstructive Pulmonary Disease (COPD). Respiratory Research 2019; 20: 152 10.1186/s12931-019-1105-z 31299954 PMC6626346

[12] Petkov DI, Liu DX, Allers C, Didier PJ, Didier ES, Kuroda MJ. Characterization of heart macrophages in rhesus macaques as a model to study cardiovascular disease in humans. Journal of Leukocyte Biology 2019; 10.1002/JLB.1A0119-017R 31287581 PMC7216974

[13] Brock M, Trekmann M, Gay RE, Gay S, Speich R, Huber LC. MicroRNA-18a enhances the interleukin-6-mediated production of the acute phase proteins fibrinogen and heptoglobin in human hepatocytes. The Journal of Biological Chemistry 2011; 286: 40142–40150. 10.1074/jbc.M111.251793 21953462 PMC3220547

[14] Tachibana S, Zhang X, Ito K, Ota Y, Cameron AM, Williams GM, et al Interluekin-6 is required for cell cycle arrest and activation of DNA repair enzymes after partial hepatectomy in mice. Cell and Bioscience 2014; 4: 6 10.1186/2045-3701-4-6 24484634 PMC3922598

[15] Wong CP, Rinaldi NA, Ho E. Zinc deficiency enhanced inflammatory response by increasing immune cell activation and inducing IL6 promoter demethylation. Molecular Nutrition and Food Research 2015; 59: 991–999. 10.1002/mnfr.201400761 25656040 PMC4425307

[16] Balkwill F. TNF-alpha in promotion and progression of cancer. TNF-alpha in promotion and progression of cancer. Cancer Metastasis Reviews 25; 2006: 409–416. 10.1007/s10555-006-9005-3 16951987

[17] Sumner DR, Ross R, Purdue E. Are there biological markers for wear or corrosion? A systemic review. Clinical Orthopaedics and Related Research 2014; 472: 3728–3739. 10.1007/s11999-014-3580-3 24668073 PMC4397751

[18] Mocellin S, Panelli MC, Wang E, Nagorsen D, Marincola FM. The Dual Role of IL10. Trends in Immunology 2004; 24: 36–43. 10.1016/s1471-4906(02)00009-1 12495723

[19] Moore KW, Malefyt R, Coffman RL, O’Garra A. Interluekin-10 and the interluekin-10 receptor. Annual Review of Immunology 2001; 19: 683–765. 10.1146/annurev.immunol.19.1.683 11244051

[20] PATROLS - https://www.patrols-h2020.eu/ - accessed 7 March 2019.

[21] Kermanizadeh A, Løhr M, Roursgaard M, Messner S, Gunness P, Kelm JM, et al Hepatic toxicology following single and multiple exposure of engineered nanomaterials utilising a novel primary human 3D liver microtissue model. Particle and Fibre Toxicology 2014; 11: 56 10.1186/s12989-014-0056-2 25326698 PMC4207326

[22] Kermanizadeh A, Jacobsen NR, Roursgaard M, Loft S, Møller P. Hepatic hazard assessment of silver nanoparticle exposure in healthy and chronically alcohol fed mice. Toxicological Sciences 2017; 158: 176–187. 10.1093/toxsci/kfx080 28453772

[23] Kermanizadeh A, Brown DM, Moritz W, Stone V. The importance of inter-individual Kupffer cell variability in the governance of hepatic toxicity in a 3D primary human liver microtissue model. Accepted in Nature Scientific Reports 2019 10.1038/s41598-019-39903-x PMC6513945 31086251

[24] Gosens I, Kermanizadeh A, Jacobsen NR, Lenz AG, Bokkers B, de Jong WH, et al Comparative hazard identification by a single dose lung exposure of zinc oxide and silver nanomaterials in mice. Plos One 2015; 10: e0126934 10.1371/journal.pone.0126934 25966284 PMC4429007

[25] Jacobsen NR, Stoeger T, van den Brule S, Saber AT, Beyerle A, Vietti G, et al Acute and subacute pulmonary toxicity and mortality in mice after intratracheal instillation of ZnO nanoparticles in three laboratories. Food and Chemical Toxicology 2015; 85: 84–95. 10.1016/j.fct.2015.08.008 26260750

[26] Vranic S, Gosens I, Jacobsen NR, Jensen KA, Bokkers B, Kermanizadeh A, et al Impact of serum as a dispersion agent for *in vitro* and *in vivo* toxicological assessments of TiO_2_ nanoparticles. Archives of Toxicology 2017; 91: 353–363. 10.1007/s00204-016-1673-3 26872950

[27] JRC nanomaterials repository - https://ec.europa.eu/jrc/sites/jrcsh/files/JRC-Nanomaterials-Repository-List-of-Representative-Nanomaterials.pdf - accessed 7 March 2019.

[28] Kermanizadeh A, Pojana G, Gaiser BK, Birkedal R, Bilaničová D, Wallin H, et al *In vitro* assessment of engineered nanomaterials using C3A cells: cytotoxicity, pro-inflammatory cytokines and function markers. Nanotoxicology 2013; 7: 301–313. 10.3109/17435390.2011.653416 22263564

[29] Septiadi D, Abdussalam W, Rodriguez-Lorenzo L, Spuch-Calvar M, Bourquin J, Petri-Fink A, et al Revealing the role of epithelial mechanics and macrophage clearance during pulmonary epithelial injury recovery in the presence of carbon nanotubes. Advanced Materials 2018; 30: e1806181 10.1002/adma.201806181 30370701

[30] Kermanizadeh A, Villadsen K, Østrem RG, Jensen KJ, Møller P, Loft S. Integrin targeting and toxicological assessment of peptide-conjugated liposome delivery systems to activated endothelial cells. Basic and Clinical Pharmacology and Toxicology 2017; 120: 380–389. 10.1111/bcpt.12692 27767251

[31] NANOGENOTOX - https://www.anses.fr/en/system/files/nanogenotox_deliverable_5.pdf - accessed 12 May 2018.

[32] Kermanizadeh A, Chauché C, Balharry D, Brown DM, Kanase N, Boczkowski J, et al The role of Kupffer cells in the hepatic response to silver nanoparticles. Nanotoxicology 2014; 8: 149–154. 10.3109/17435390.2013.866284 24344730

[33] Schiwon M, Franken L, Kurts C, Engel DR. Crosstalk between sentinel and helper macrophages permits neutrophil migration into infected uroepithelium. Cell 2014; 156: 456–468. 10.1016/j.cell.2014.01.006 24485454 PMC4258064

[34] Dabrowski MP, Stankjewicz W, Plusa T, Chcialowski A, Szmigielski. Competition of IL-1 and IL-1ra determines lymphocyte response to delayed stimulation with PHA. 2001. Mediators of Inflammation 2001; 10: 101–107. 10.1080/09629350124376 11545246 PMC1781706

[35] Gonnord P, Angermann BR, Sadtler K, Gombos E, Chappert P, Meier-Schellersheim M, et al A hierarchy of affinities between cytokine receptors and the common gamma chain leads to pathway cross-talk. Science Signaling 2018; 11: 544. 10.1126/scisignal.aal1253 29615515

[36] Brown DM, Donaldson K, Stone V. Nuclear translocation of Nrf2 and expression of antioxidant defence genes in THP-1 cells exposed to carbon nanotubes. Journal of Biomedical and Nanotechnology 2010; 6: 224–233. 10.1166/jbn.2010.1117 21179939

[37] Cao Z, Fang Y, Lu Y, Qian F, Ma Q, He M, Pi H, Yu Z, Zhou Z. Exposure to nickel oxide nanoparticles induces pulmonary inflammation through NLRP3 inflammasome activation in rats. International Journal of Nanomedicine 2016; 11: 3331–3346. 10.2147/IJN.S106912 27524893 PMC4965228

[38] Hamilton R Jr, Wu Z, Mitra S, Shaw PK, Holian A. Effect of MWCNT size, carboxylation, and purification on *in vitro* and *in vivo* toxicity, inflammation and lung pathology. Particle and Fibre Toxicology 2013; 10: 57 10.1186/1743-8977-10-57 24225053 PMC3830505

[39] Jessop F, Holian A. Extracellular HMBG1 regulates multi-walled carbon nanotube-induced inflammation *in vivo*. Nanotoxicology 2015; 9: 365–372. 10.3109/17435390.2014.933904 24983895 PMC4763602

